# Optimization of Alkaline and Dilute Acid Pretreatment of Agave Bagasse by Response Surface Methodology

**DOI:** 10.3389/fbioe.2015.00146

**Published:** 2015-09-23

**Authors:** Abimael I. Ávila-Lara, Jesus N. Camberos-Flores, Jorge A. Mendoza-Pérez, Sarah R. Messina-Fernández, Claudia E. Saldaña-Duran, Edgar I. Jimenez-Ruiz, Leticia M. Sánchez-Herrera, Jose A. Pérez-Pimienta

**Affiliations:** ^1^Department of Chemical Engineering, Universidad Autónoma de Nayarit, Tepic, Mexico; ^2^Department of Engineering in Environmental Systems, Instituto Politécnico Nacional, Mexico City, Mexico; ^3^Cuerpo Académico de Sustentabilidad Energética, Universidad Autónoma de Nayarit, Tepic, Mexico; ^4^Food Technology Unit, Universidad Autónoma de Nayarit, Tepic, Mexico

**Keywords:** agave bagasse, high solids, biomass pretreatment, optimization, characterization

## Abstract

Utilization of lignocellulosic materials for the production of value-added chemicals or biofuels generally requires a pretreatment process to overcome the recalcitrance of the plant biomass for further enzymatic hydrolysis and fermentation stages. Two of the most employed pretreatment processes are the ones that used dilute acid (DA) and alkaline (AL) catalyst providing specific effects on the physicochemical structure of the biomass, such as high xylan and lignin removal for DA and AL, respectively. Another important effect that need to be studied is the use of a high solids pretreatment (≥15%) since offers many advantaged over lower solids loadings, including increased sugar and ethanol concentrations (in combination with a high solids saccharification), which will be reflected in lower capital costs; however, this data is currently limited. In this study, several variables, such as catalyst loading, retention time, and solids loading, were studied using response surface methodology (RSM) based on a factorial central composite design of DA and AL pretreatment on agave bagasse using a range of solids from 3 to 30% (w/w) to obtain optimal process conditions for each pretreatment. Subsequently enzymatic hydrolysis was performed using Novozymes Cellic CTec2 and HTec2 presented as total reducing sugar (TRS) yield. Pretreated biomass was characterized by wet-chemistry techniques and selected samples were analyzed by calorimetric techniques, and scanning electron/confocal fluorescent microscopy. RSM was also used to optimize the pretreatment conditions for maximum TRS yield. The optimum conditions were determined for AL pretreatment: 1.87% NaOH concentration, 50.3 min and 13.1% solids loading, whereas DA pretreatment: 2.1% acid concentration, 33.8 min and 8.5% solids loading.

## Introduction

Lignocellulosic biomass is the most abundant renewable carbohydrate source in the world and it is proposed to dominate the biofuel production in the future (Avci et al., 2013). Mainly composed by cellulose, hemicellulose, and lignin, their organization and interaction between these polymeric structures, the plant cell wall is naturally recalcitrant to biological degradation (da Costa Sousa et al., 2009). A pretreatment step is fundamental to alter the structure of cellulosic biomass to make cellulose more accessible to the enzymes that convert the carbohydrate polymers into fermentable sugars (Mosier et al., 2005).

Many options exist for pretreatment of biomass, increase saccharification efficiency and improve the yields of monomerics sugars; the leading examples use liquid catalysts, such as sulfuric acid, ammonia, ionic liquid, or water, which penetrate the cell wall and alter its chemistry and ultrastructure (Dadi et al., 2006; Chundawat et al., 2011).

Recently, agave bagasse (AGB) byproduct of the Tequila industry that represent 40% of the harvested plant, with an annual generation in Mexico of about 1.12 kg × 10^8^ kg has been studied for biomass conversion using different pretreatment approaches, such as ionic liquid (Perez-Pimienta et al., 2013) and organosolv (Caspeta et al., 2014). Moreover, AGB was also been used with acid and enzymatic hydrolysis followed by a fermentation step using a native microorganism (Pichia caribbica UM-5) obtaining ~57% of theoretical ethanol (w/w) (Saucedo-Luna et al., 2011) or for the production of n-butanol and ethanol from different Agave species (Mielenz et al., 2015).

Dilute acid (DA) and alkaline (AL; NaOH) are among the most extensively studied biomass pretreatments in different feedstocks, such as grasses, agricultural residues, and woods (Kumar et al., 2009; Xu et al., 2010; Sathitsuksanoh et al., 2013; Zhang et al., 2014). The mode of action of the DA pretreatment typically use sulfuric acid that removes hemicellulose in a great extent improving the enzyme accessibility to cellulose which its effectiveness depends on the acid concentration and temperature applied during the process, however, if severe conditions are applied several degradation products are formed, mainly furfural, 5-hydroxymethylfurfural, phenolic acids and aldehydes, levulinic acid, and other aliphatic acids, which can inhibit both, enzymatic hydrolysis and fermentation (Mosier et al., 2005; da Costa Sousa et al., 2009). On the other hand, ALs pretreatment uses AL catalyst, such as sodium hydroxide, which are effective depending on the lignin content on the biomass, increasing cellulose digestibility through lignin solublization/removal, exhibiting minor cellulose and hemicellulose solubilization than acid or hydrothermal processes (Avira et al., 2010).

In recent years, the need to investigate the use of high solids loading (≥ 15%) in biomass pretreatment has increase hence offers many advantaged over lower solids loadings, including increased sugar and ethanol concentrations, which will be reflected in lower capital costs (Modenbach and Nokes, 2012; Li et al., 2013); however, this data is currently limited for DA and AL pretreatments in AGB (Hernández-Salas et al., 2009; Saucedo-Luna et al., 2011).

In the present manuscript, optimization of DA and AL pretreatment strategies for conversion of AGB to sugars using a central composite design (CCD) for response surface methodology (RSM) was studied. The objective of this study was to identify the optimum process conditions for the selected operating variables namely catalyst concentration, retention time, and solid loading for the maximum production of fermentable sugars. Furthermore, the untreated and selected samples from both pretreatments were characterized by calorimetric techniques (TGA), fluorescence and energy dispersive X-ray spectroscopy (EDS), and scanning electron microscopy (SEM).

## Materials and Methods

The biomass used in this study was obtained from Destilería Rubio, a Tequila plant from western Mexico. The AGB was harvested in August 2014. The biomass was milled with a Thomas-Wiley Mini Mill fitted with a 40-mesh screen (Model 3383-L10 Arthur H. Thomas Co., Philadelphia, PA, USA) and stored at 4°C in a sealed plastic bag. Cellic^®^ CTec2 (Cellulase complex for degradation of cellulose) and HTec2 (Endoxylanase with high specificity toward soluble hemicellulose) were a gift from Novozymes (Davis, CA, USA).

### Experimental design

Optimization of processing conditions for fermentable sugars recovery was studied using a factorial CCD of RSM. The independent variables were catalyst concentration, residence time, and solids loading. The experimental data were fit using Eq. (1), a low-order polynomial equation to evaluate the effect of each independent variable to the response, which was later analyzed to obtain the optimum process conditions (Tan et al., 2011). In this study, a polynomial quadratic equation was employed as follows:

(1) $$y=\beta_{0}+\sum_{i=1}^{3}\beta_{i}X_{i}+\sum_{i=1}^{3}\beta_{\mathrm{ii}}{X_{i}}^{2}+\sum_{i=1}^{3}\sum_{i=1}^{3}\beta_{\mathrm{ii}}X_{i}X_{j}$$

where *y* is the response, *X_i_* and *X_j_* are independent variables, β_0_ is the constant coefficient, β*_i_* is the *i*th linear coefficient, β*_ii_* is the quadratic coefficient, and β*_ij_* is the *ij*th interaction coefficient. CCD consists of 2*^k^* factorial points, 2*k* axial points (± α), and six central points, where *k* is the number of independent variables. Each of the variables were investigated at five coded levels (−α, −1, 0, 1, α), as listed in Table 1, and the complete experimental design matrix for this study is shown in Table 2. For each pretreatment (DA and AL), a total of 20 experiments per pretreatment were carried out, including eight per factorial design, six for axial points and six repetitions at the central point.

**Table 1:** **Levels of the pretreatment condition variables tested in the CCD**.

Variable	Unit	Coding	Coded level				
			−α^a^	−1	0	1	+α^a^
Catalyst concentration	% (w/w)	A	0.15	0.73	1.58	2.42	3.00
Residence time	Min	B	15.00	30.20	52.50	74.80	90.00
Solids loading	% (w/w)	C	3.00	8.47	16.50	24.53	30.00

*^a^α (axial distance) = ^4^√*N*, where *N* is the number of experiments of the factorial design. In this case, 1.6818*.

**Table 2:** **Experimental design matrix of CCD and corresponding results (sugars and solids recovery)**.

Run	Experimental variables			Solids recovery (%)		TRS yield (mg/g biomass)	
	Catalyst concentration, A (%, w/w)	Retention time, B (min)	Solids loading, C (%, w/w)	AL	DA	AL	DA
1	1.58	15.00	16.50	70.3	71.9	506.1	419.3
2	2.42	30.20	8.47	74.3	58.5	476.9	391.3
3	0.73	30.20	8.47	75.5	83.0	447.5	410.9
4	2.42	30.20	24.53	72.3	66.8	468.2	371.6
5	0.73	30.20	24.53	84.3	82.7	415.6	385.7
6	0.15	52.50	16.50	86.2	80.8	360.3	339.7
7	1.58	52.50	3.00	65.5	57.6	513.6	457.2
8	3.00	52.50	16.50	60.7	58.3	457.9	364.5
9	1.58	52.50	30.00	80.6	68.7	421.0	399.7
10	2.42	74.80	8.47	63.4	54.4	460.5	360.5
11	0.73	74.80	8.47	72.4	77.3	460.1	428.0
12	2.42	74.80	24.53	74.6	65.3	453.7	353.7
13	0.73	74.80	24.53	87.6	86.1	437.2	409.0
14	1.58	90.00	16.50	72.8	70.4	521.5	397.9
15	1.58	52.50	16.50	76.2	68.6	532.8	394.4
16	1.58	52.50	16.50	76.7	71.6	517.1	415.2
17	1.58	52.50	16.50	78.2	68.8	497.2	443.8
18	1.58	52.50	16.50	79.3	70.6	521.3	431.5
19	1.58	52.50	16.50	79.9	69.5	502.7	439.4
20	1.58	52.50	16.50	77.8	70.4	511.6	435.9
Untreated	–	–	–	100		135.1	

*AL, alkaline pretreatment; DA, dilute acid pretreatment*.

### Alkaline pretreatment

A NaOH solution at a specific concentration were placed in a serum bottle and mixed with AGB using a glass rod, forming a slurry at with a precise biomass concentration and the pretreatment was performed in autoclave conditions (121°C and ~15 psi) during the appropriate time according to Table 1 (Xu et al., 2010). Pretreated biomass was recovered by filtration and washed with 400 mL of distilled water to remove excess alkali and dissolved byproducts. All experiments were conducted in triplicate.

### Dilute acid pretreatment

The DA pretreatment with H_2_SO_4_ was conducted using the appropriate acid concentration and solids loading referred to Table 1 at 130°C and 20 psi in an autoclave for a specific time (Sathitsuksanoh et al., 2013). After DA, the hydrolyzate was separated by filtration and the pretreated AGB was washed with 400 mL of distilled water prior to enzymatic hydrolysis. All experiments were conducted in triplicate.

### Scanning electron microscopy

The morphology of untreated and selected pretreated AGB solids was analyzed using a high resolution SEM by a JEOL JSM-7800F equipment. The representative images were acquired with a 1 kV accelerating voltage and analysis using 20 kV.

### Confocal fluorescent microscopy

The confocal fluorescent microscope images of untreated and selected pretreated AGB samples were taken using a Carl Zeiss LSM 710 NLO with two laser sources (405 and 633 nm). To demonstrate the microstructure based on the distribution of lignin (autofluorescence) and cellulose, all samples were labeled with Calcofluor white stain (0.1%) for 5 min, subsequently were washed four times using distilled water and allowed to dry in the dark until analysis under the confocal microscope.

### DSC and TGA analysis

A differential scanning calorimeter (Pyris 1) from Perkin Elmer was employed with an argon atmosphere in the range of 50–450°C, at 10°C/min ramp. DSC curves were obtained with 3.3 mg. The TGA curves were obtained using around 3.8 mg of AGB as initial sample mass. The samples was tested in a SETARAM thermal analysis instrument, with temperature range of 50–800°C and heating rate of 10°C/min in argon atmosphere. Untreated and selected pretreated samples were measured by DSC and TGA.

### Biomass porosimetry

Nitrogen porosimetry (ASAP 2406) from Mca-Micromeritics was employed to measure the surface area, pore volume and pore size distribution of the untreated and selected pretreated AGB with the following methods from ASTM: ASTM D-3663(R2008), ASTM D-4222-03(R2008), and ASTM D-4641-12(R2008). Samples were degasified at 120°C.

### Enzymatic saccharification

The saccharification was carried out using commercially available Cellic^®^ CTec2 and HTec2 enzyme mixtures of untreated and pretreated AGB samples, which was conducted at 55°C and 150 rpm in 50 mM citrate buffer (pH of 4.8). A 3% biomass loading was used, likewise, untreated AGB were run concurrently with the pretreated samples to eliminate potential differences in temperature history or enzyme loading. The enzyme concentrations of CTec2 and HTec2 were set at 35 FPU/g biomass and 60 CBU/g biomass, respectively. All assays were performed in triplicate.

### DNS assay

The total reducing sugar (TRS) yield of the final hydrolyzate calculated as mg sugar/g biomass was determined by DNS assay (Miller, 1959) on a DTX 880 Multimode Detector (Beckman Coulter, CA, USA) at 550 nm with solutions (0–10 g/L) of d-glucose in water as calibration standards. All assays were performed in triplicate.

### Statistical analysis

Analysis of experimental CCD results was carried out with the software Design-Expert 7.1.5 (Stat-Ease, Minneapolis, MN, USA). Each coefficient in Eq. (1) was calculated and the possible interaction effects of the process variables on the response were obtained. Their significance was checked by variance analysis (ANOVA) of experimental results.

## Results and Discussion

### Biochemical composition analysis of untreated agave bagasse

By following, the National Renewable Energy Laboratory (NREL, Denver, CO, USA) protocols, the composition of untreated AGB in dry basis was 41.5% glucan, 20.3% xylan, 17.0% insoluble lignin, 3.8% soluble lignin, and 5.4% ash, which is consistent with other reported values (Davis et al., 2011; Perez-Pimienta et al., 2013). Glucan and xylan correspond to 61.8% of the total carbohydrates in the AGB.

### Model development

The experimental data were first analyzed, in order to obtain second-order polynomial equations including terms of interaction between the experimental variables using Design-Expert software and the following models for AL and DA pretreatment describes the TRS yield (mg sugar/g biomass) in terms of coded parameters and actual parameters are based on the statistical analysis of the experimental data shown in Table 2.

The final equations for AL pretreatment were as follows:

(2) $$\begin{array}{l} \text{TRS yield}=513.35+21.08\times A+3.57\times B-16.87\times C-9.95\times \mathrm{AB}+4.87\times \mathrm{AC}+\\ \;1.67\times \mathrm{BC}-38.44\times A^{2}-2.52\times B^{2}-18.14\times C^{2} \end{array}$$

(3) $$\begin{array}{l} \text{TRS yield}=\text{277.0937}+\text{203.1844}*\text{NaOH}+\text{1.0059}*\text{Time}+5.6903*\text{Solids}-\\ \;0.4313*\text{NaOH}*\text{Time}+0.7172*\text{NaOH}*\text{Solids}+0.0076*\text{Time}*\text{Solids}-\\ \;53.8367*\text{NaO}{\text{H}}^{\text{2}}-0.0034*\text{Tim}{\text{e}}^{\text{2}}-0.2813*\text{Solid}{\text{s}}^{\text{2}} \end{array}$$

**Figure 1:**
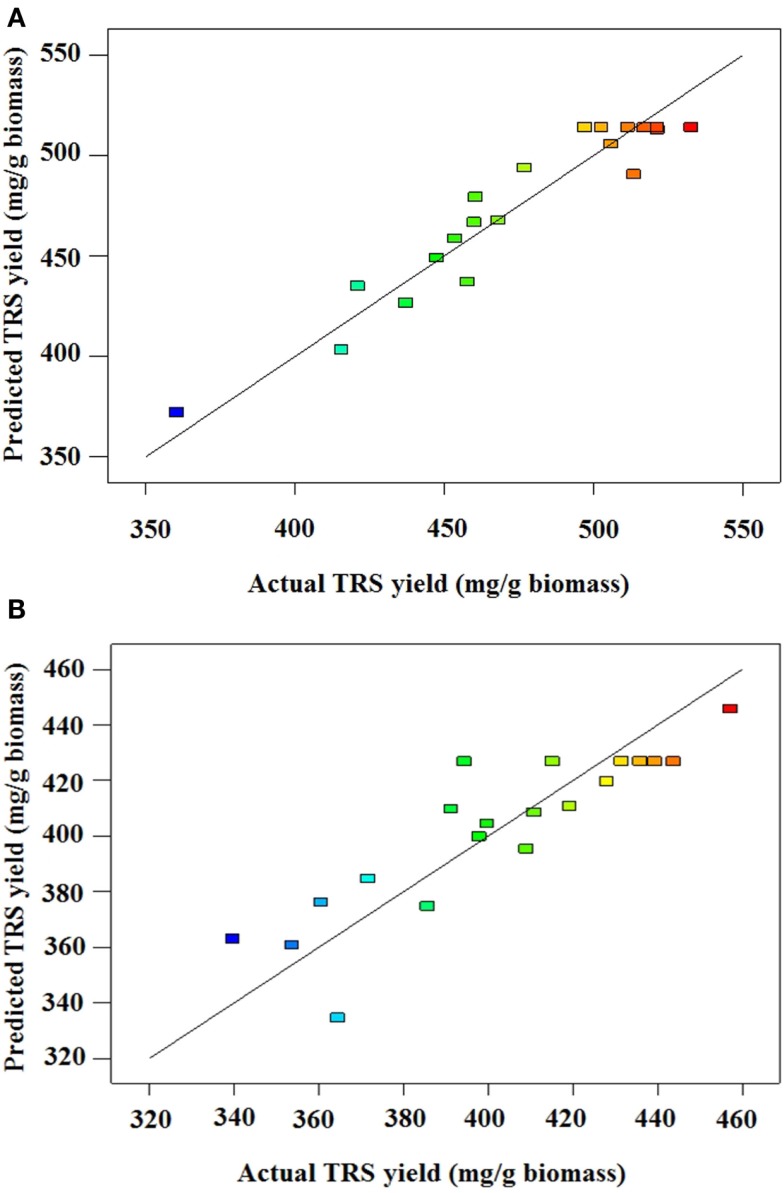
**(A)** Predicted vs. actual TRS yield of alkaline-pretreated AGB. **(B)** Predicted vs. actual TRS yield of dilute acid pretreated AGB.

In the same way, the final equations for DA pretreatment were as follows:

(4) $$\begin{array}{l} \text{TRS yield}=427.27-5.87\times A+0.26\times B-12.80\times C-13.65\times \mathrm{AB}+2.19\times \mathrm{AC}+\\ \;2.92\times \mathrm{BC}-27.48\times A^{2}-11.45\times B^{2}-0.62*C^{2} \end{array}$$

(5) $$\begin{array}{lll} \text{TRS yield}=305.8687+137.0487\times \text{Acid}+2.1816\times \text{Time}-2.4166\times \text{Solids}-\\ \;0.5917\times \text{Acid}\times \text{Time}+0.3231\times \text{Acid}\times \text{Solids}+0.0133\times \text{Time}\times \text{Solids}-384832\times & & \\ \text{ Aci}{\text{d}}^{2}-0.0154\times \text{Tim}{\text{e}}^{2}-0.0097\times \text{Solid}{\text{s}}^{2} \end{array}$$

where A, B, and C are catalyst concentration (NaOH for AL and H_2_SO_4_ for DA), retention time and solids loading, respectively. An analysis of variance (ANOVA) was performed to test the significance of the developed model and the results are presented for AL and DA pretreatment in Tables 3 and 4, respectively. If a *p*-value (also known as the Prob > *D*-value) is lower than 0.05 a model in considered significant, indicating only a 5% chance that their respective model could occur due to noise. For both pretreatments, their models effectively describes the response nevertheless the AL pretreatment model have a lower *p*-value (0.0003) than the DA pretreatment model (0.0247). In addition, the Prob > *F* values for each model term in AL pretreatment suggest that A, C, and A^2^, meanwhile for DA pretreatment suggest that only A^2^ are the model terms that have significant effects on the TRS yield. To determine the suitability of the model, the lack of fit test was used, which indicated an insignificant lack of fit with an *F*-value of 0.1393 and 0.3009 for AL and DA pretreatment, respectively. The coefficient of determination (*R*^2^) of the pretreatment models was 0.9151 for AL and 0.7270 and for DA, implying a good and average correlation between the observed and predicted values of AL and DA respectively, as shown in Figures 1A,B. Finally, the quadratic models developed for AL and DA pretreatment are appropriate for predicting TRS yield under different pretreatment conditions within the range used in the present study.

**Table 3:** **ANOVA table for the quadratic model of alkaline pretreatment**.

Source	Sum of squares	DF	Mean square	*F*-value	Prob > *F*	
Model	34291.73	9	3810.19	11.97	0.0003	Significant
A	5600.42	1	5600.42	17.59	0.0018	
B	97.31	1	97.31	0.31	0.5925	
C	3578.16	1	3578.16	11.24	0.0073	
AB	528.42	1	528.42	1.66	0.2266	
AC	189.46	1	189.46	0.60	0.4583	
BC	14.85	1	14.85	0.047	0.8333	
A^2^	21479.83	1	21479.83	67.48	<0.0001	
B^2^	40.82	1	40.82	0.13	0.7277	
C^2^	4734.21	1	4734.21	14.87	0.0032	
Residual	3183.28	10	318.33			
Lack of fit	2351.53	5	470.31	2.83	0.1393	Not significant
Pure error	831.75	5	166.35			

*DF, degree of freedom*.

**Table 4:** **ANOVA table for the quadratic model of dilute acid pretreatment**.

Source	Sum of squares	DF	Mean square	*F*-value	Prob > *F*	
Model	15632.82	9	1736.98	3.79	0.0247	Significant
A	434.69	1	434.69	0.95	0.3529	
B	0.51	1	0.51	0.001	0.9739	
C	2059.98	1	2059.98	4.50	0.0599	
AB	994.66	1	994.66	2.17	0.1713	
AC	38.45	1	38.45	0.084	0.7779	
BC	45.60	1	45.60	0.100	0.7588	
A^2^	10975.31	1	10975.31	23.96	0.0006	
B^2^	841.41	1	841.41	1.84	0.2051	
C^2^	5.61	1	5.61	0.012	0.9140	
Residual	4580.07	10	458.01			
Lack of fit	2843.15	5	568.63	1.64	0.3009	Not significant
Pure error	1736.92	5	347.38			

*DF, degree of freedom*.

### Effect of pretreatment conditions on solids recovery

The highest solids recovery for AL and DA was obtained in the same run (13) with 87.6 and 86.1%, respectively, with experimental conditions of 0.73% catalyst concentration, 74.8 min and 24.53% solids loading. On the other hand, the lowest solids recovery for AL pretreatment of 60.7% was obtained during run 8 (3.00% catalyst concentration, 52.5 min and 16.5% solids loading), while for DA pretreatment was 54.4% with run 10 using 2.42% catalyst concentration, 74.8 min and 8.47% solids loading. The difference between low and high solids recovery, which represents process severity are 26.9 and 31.7% for AL and DA pretreatment, respectively.

### Effect of pretreatment catalyst concentration and retention time

**Figure 2:**
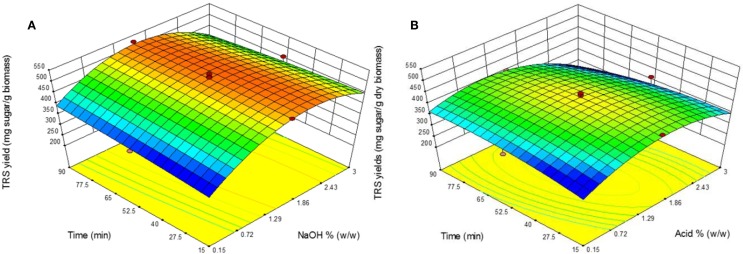
**Response surface plots showing the effects of time and catalyst concentration for (A) alkaline pretreatment and (B) dilute acid pretreatment**.

The effect of catalyst concentration and retention time in AL and DA pretreatment on TRS yield during enzymatic saccharification using 3% biomass loading of are shown in Figure 2. By means of pretreatment shorter retention times and catalyst concentration, the TRS yield became lower and the same applies to longer times and high catalyst concentration for both AL and DA pretreatment.

However, for AL pretreatment from 1.58 to 2.43% NaOH a TRS yield above ~460 mg sugar/g biomass is obtained within the study range of 15–90 min. In the other hand, in DA pretreatment a more distributed region is shown where the highest TRS yields was obtained at the central design points with a relatively shorter differences between the highest yield that occurred in run 7 (457 mg/g biomass) and an average of the central data points (433 mg/g biomass).

### Effect of pretreatment catalyst concentration and solid loading

**Figure 3:**
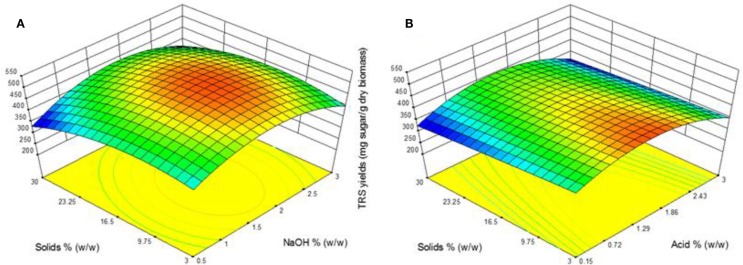
**Response surface plots showing the effects of solid loading and catalyst concentration for (A) alkaline pretreatment and (B) dilute acid pretreatment**.

The response surface plots presents the effect of catalyst concentration and solid loading on TRS yield of both AL and DA pretreatment is displayed in Figure 3. One area for AL pretreatment is clearly defined showing the highest TRS yield region in the middle range of both parameters. A TRS yield above 500 mg/g biomass is obtained in the range of 1.1–2.3% NaOH and solid loading between 4 and 20%. These results are supported with previous reports in AL pretreatment where using the same temperature conditions (121°C), moderate NaOH concentration (1%) and time (30–60 min), which achieved the highest TRS yield (Wang et al., 2010; Xu et al., 2010). During DA pretreatment a clear region where a TRS yield above 430 mg/g biomass was reached within the range of 0.7–2% acid and a solid loading of 3–15%. It is noticeable that such differences between the TRS yields were obtained from the highest experimental runs from both pretreatments at ~533 mg/g biomass from run 15 in AL and ~457 mg/g biomass from run 7 in DA. This differences are encounter from the objective of each pretreatment, which in the case of AL pretreatment is lignin removal whereas for DA pretreatment xylan removal is the main effect, as consequence a lower TRS yield should be obtained as there is lower xylan available as a substrate for the enzymes to be reacted into xylose causing a lower total TRS yield.

### Optimization of pretreatment conditions

In both of the evaluated pretreatment processes (AL and DA), a lower catalyst concentration, shorter time and high solids loading if preferred to obtain an optimum TRS yield. The optimum catalyst concentration, retention time and solid loading were found to be for AL pretreatment of 1.87% NaOH concentration, 50.3 min and 13.1% solids loading, while DA pretreatment were 2.1% acid concentration, 33.8 min and 8.5% solids loading. For AL pretreatment, an 18% increase in NaOH concentration, 4% reduction in retention time and 20% reduction of solids loading, whereas for DA pretreatment, 33% increase in acid concentration, 35.6% reduction in retention time and 283% increase of solids loading and when comparing the optimum conditions with the experimental conditions (Run 7, Table 2) that gave the highest yields.

### Thermogravimetric and differential scanning calorimetry analysis

**Figure 4:**
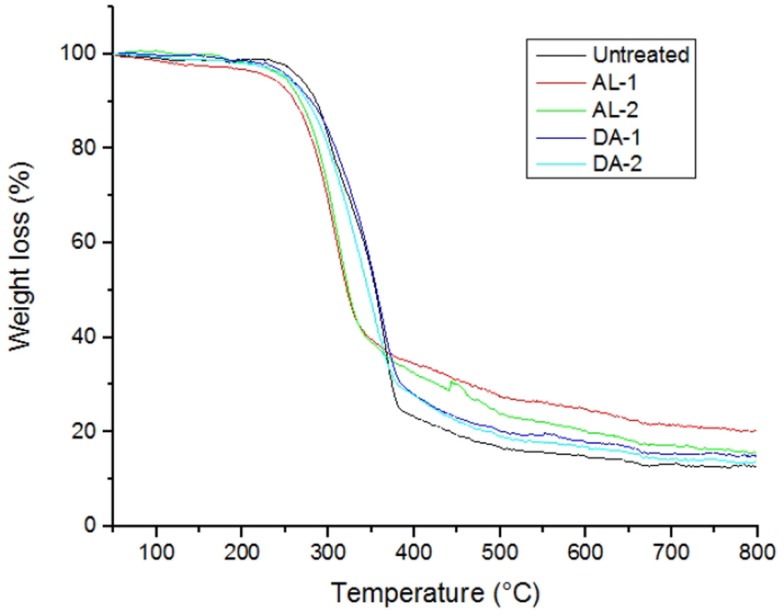
**TG curves of untreated and selected pretreated samples**. AL, alkaline and DA, dilute acid.

**Figure 5:**
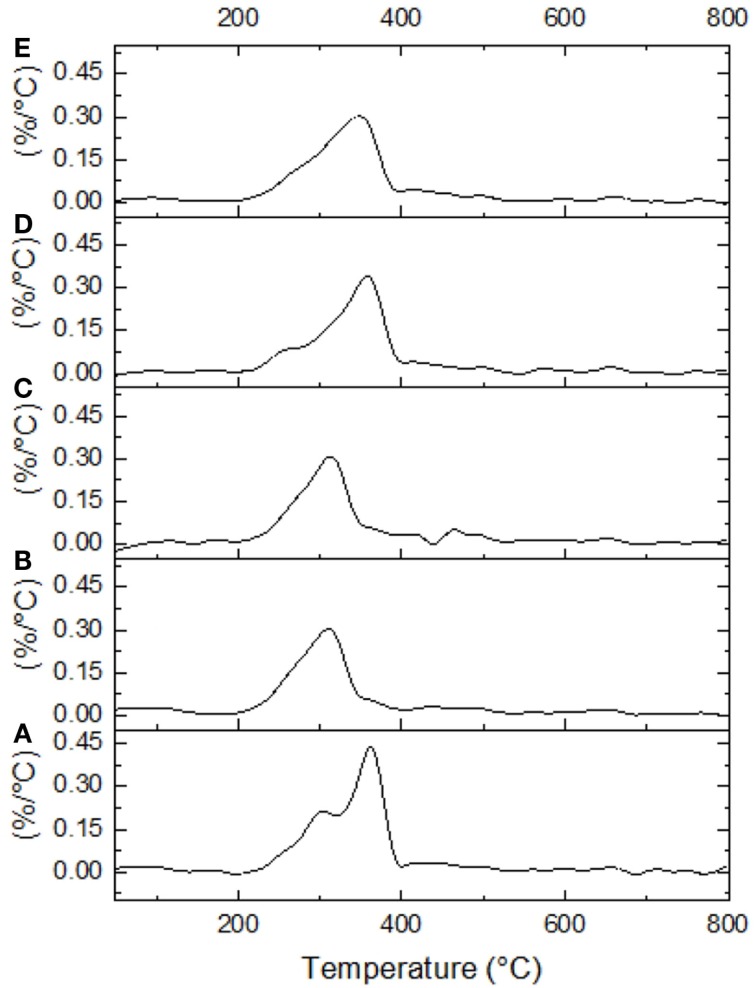
**Differential TGA plots are shown for untreated and selected pretreated samples**. **(A)** Untreated AGB, **(B)** AL-1, **(C)**. AL-2, **(D)** DA-1, and **(E)** DA-2.

Untreated and selected pretreated AGB samples were thermogravimetrically analyzed to compare degradation characteristics in terms of pretreatment. Two samples were selected for TGA analysis for each pretreatment, named AL-1 and DA-1 corresponding to experimental run 8, in addition to AL-2 and DA-2 corresponding to experimental run 16 (one of the CCD points). Figure 4 shows standards weight loss plots, while in Figure 5 the differential TGA plots of the untreated and pretreated AGB samples are shown. All samples exhibit three decomposition regions with some initial weight loss from 50 to 125°C (mainly due to moisture evaporation). Up to 200°C, the samples presented thermal stability. The decomposition temperature (*T*_d_) decrease for both AL and AL pretreated samples as compared to the untreated AGB, shown in Table 5. In both of the analyzed pretreatment the lowest values correspond to AL-1 (run 8 sample). These results indicate that AL pretreatment reduced the activation energy that is needed to decompose the AGB in a higher extent than DA pretreatment by deconstructing the tight plant cell wall structures. AL-pretreated AGB samples obtained a lower *T*_d_ value when compared to an ionic liquid treated AGB from a recent report (310 vs. 347°C) (Perez-Pimienta et al., 2015). Thermal depolymerization of hemicelluloses and the cleavage of glycosidic linkages of cellulose occurs in the region of 220–300°C, while lignin decomposition extended to the whole temperature range, from 200 until 700°C, due to different activities of the chemical bonds present on its structure and the degradation of cellulose taken place between 275 and 400°C (Deepa et al., 2011). The final decomposition stage for all samples was completed above 400°C, where a weight loss due to thermolysis of carbon containing residues does take place (Fisher et al., 2002). DSC curves of untreated AGB and selected samples from AL and DA (Figures S1–S3 in Supplementary Material) with two endothermic peaks observed and Table S1 in Supplementary Material summarizes those events. The first thermal is shown below 200°C with low energy between 5.3 and 13.9 J/g°C, where the untreated AGB present the onset temperature at 83°C (8.6 J/g°C), while the AL-4 (run 16 of AL pretreatment) achieved 13.9 J/g°C, whereas for DA the highest energy event was at 12.2 J/g°C with DA-1 (run 8) that employed a 3% acid loading. A similar peak was obtained with an IL-treated AGB sample where the untreated sample showed a dehydration peak at 89°C (Perez-Pimienta et al., 2015). In the other hand, the second thermal event presents a high energy peak for all samples with ΔH in the range of 120–627 J/g°C and temperature above 262 up to 415°C. AL pretreatment achieved its highest energy with run 16 (AL-4) with a peak at 335°C (627 J/g°C), whereas the evaluated DA-pretreated samples was with run 9 (AL-2) at 358°C and 296 J/g, so when compared to the untreated sample it is clear that a pretreated offers a reduction in terms of calorific value turning them into a more digestible biomass.

**Table 5:** **Decomposition *T*_d_ temperatures for untreated and pretreated AGB**.

Property	Pretreatment				
	Untreated	AL-1	AL-2	DA-1	DA-2
*T*_d_ (°C)	366	310	317	360	353

### Scanning electron and confocal fluorescence microscopy

**Figure 6:**
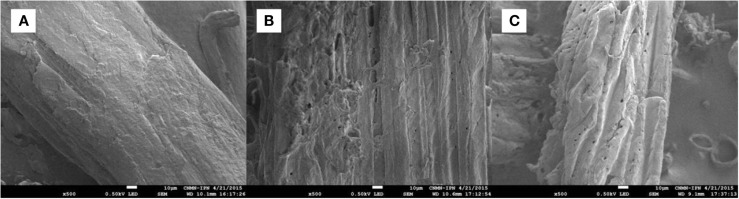
**SEM images of AGB samples: (A) untreated, (B) alkaline pretreated, and (C) dilute acid pretreated**.

The SEM images of untreated and pretreated samples (run 16 sample for both AL and DA pretreatment) were taken at 500× (Figure 6). Untreated AGB (Figure 6A) presents an intact structure without degradation, otherwise AL pretreatments dissolves lignin disrupting the biomass, besides of the increase of pore quantity as can be observed in Figure 6B. Finally, DA pretreatment disrupts the lignocellulosic structure by mainly dissolving hemicellulose, hence, major microfibrous cellulose structures remain (Figure 6C) and some lignin or lignin–carbohydrate complexes may be condensed on the surface of the cellulose fibers.

Elements content of untreated and pretreated AGB (run 16 from AL and DA pretreatment) are presented in Table 6. In the untreated AGB, C and O accounts for a 98.5% of the totals mass fraction remaining only 1.4% of Ca, these attributable to calcium oxalate (CaC_2_O_4_) crystals in considerable quantities along the surface of the plant cell wall as referred in a previous paper (Perez-Pimienta et al., 2015). In contrast, the DA-treated AGB the available Ca was removed during the process at these conditions (1.58% acid concentration, 52.5 min and 16.5% solids loading). Nonetheless, this Ca removal does not occurred in the AL-treated sample where a small amount of Na (1.2%) was found, possibly, as a result of some of the alkali was converted to irrecoverable salts and/or incorporated into the biomass.

**Table 6:** **Elements content of untreated and pretreated agave bagasse measured by EDS spectroscopy**.

Element	Untreated		AL		DA	
	Mass fraction (%)	Atomic mass fraction (%)	Mass fraction (%)	Atomic mass fraction (%)	Mass fraction (%)	Atomic mass fraction (%)
C	51.1 ± 0.9	58.6 ± 0.7	51.7 ± 1.7	59.4 ± 1.7	60.6 ± 5.6	67.2 ± 5.2
O	47.5 ± 0.5	40.9 ± 0.6	45.2 ± 2.2	39.0 ± 2.1	39.4 ± 5.6	32.8 ± 5.2
Ca	1.4 ± 0.4	0.5 ± 0.1	1.9 ± 0.9	1.2 ± 0.5	–	–
Na	–	–	1.2 ± 0.7	0.4 ± 0.3	–	–
Total	100.0	100.0	100.0	100.0	100.0	100.0

**Figure 7:**
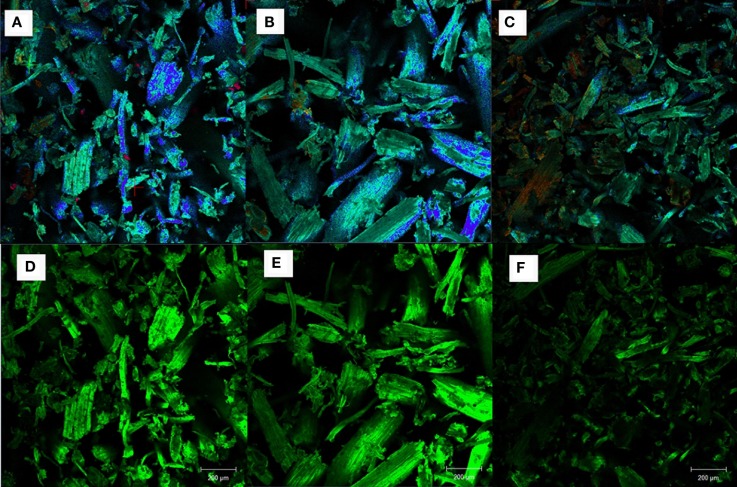
**Confocal fluorescence images of AGB samples: (A,D) untreated, (B,E) alkaline pretreated, and (C,F) dilute acid pretreated**.

Confocal fluorescence microscopy was used to investigate the surface morphologies of untreated and pretreated AGB (run 16 from AL and DA pretreatment) as presented in Figures 7A–F. When compared to the untreated AGB, only the DA-pretreated sample show a significant reduction in the fluorescence signal intensity in cell walls (lignin is represented with a green signal and cellulose with a blue signal), while the AL-treated sample presents only a slight reduction.

### Effect of pretreatment on biomass porosimetry

Pretreatment can affect the cellulose accessibility and is often accompanied by variation in the surface area. Surface area, pore volume, and pore average diameter were measured using the Brunauer–Emmett–Teller (BET) method by argon adsorption, which relates the gas pressures to the volume of gas adsorbed, although might not be directly associated to enzyme accessibility since the size differences between argon molecules and enzymes (Li et al., 2013). Table 7 summarizes surface area, pore volume and pore average diameter of untreated and run 16 (one of the CCD points from both AL and DA-pretreated AGB). When compared to the untreated samples an increment in the surface area is noticeable from 0.6 up to 1.1 m^2^/g. This is consistent with the changes in the SEM images upon AL and DA pretreatment described above. However, the pore volume of all samples (untreated and pretreated) presents a negligible difference close to 0.0008 cm^3^/g, whereas a reduction in the pore average diameter is obtained in the pretreated samples.

**Table 7:** **Comparison of porosimetry parameters in untreated and pretreated AGB**.

	Surface area (m^2^/g)	Pore volume (cm^3^/g)	Pore average diameter (A)
Untreated	0.6	0.0020	137.7
AL	0.9	0.0023	107.7
DA	1.1	0.0028	106.7

## Conclusion

The effects of catalyst concentration, retention time and solids loading in terms of TRS yield of AL and DA pretreatment in AGB were investigated. This study demonstrated that AGB is a promising biofuel feedstock that can achieved high sugar yields using both DA and AL pretreatment. For both pretreatments, a model was generated with a high correlation obtained from actual TRS data. Furthermore, the results indicate that TRS yield was enhanced by catalyst concentration and solid loading, but longer retention times does not. Both pretreatment increase porosity and surface area, but AL pretreatment achieved a lower decomposition temperature. Finally, RSM was also used to optimize the pretreatment conditions for maximum TRS yield. The optimum conditions were determined for AL pretreatment: 1.87% NaOH concentration, 50.3 min, and 13.1% solids loading, whereas DA pretreatment: 2.1% acid concentration, 33.8 min, and 8.5% solids loading. Finally, fuel synthesis studies should be performed in the sugars obtained using the best conditions for both pretreatments in order to obtain significant data for a scale-up process.

## Conflict of Interest Statement

The authors declare that the research was conducted in the absence of any commercial or financial relationships that could be construed as a potential conflict of interest.

## Supplementary Material

The Supplementary Material for this article can be found online at http://journal.frontiersin.org/article/10.3389/fbioe.2015.00146
