# Supplementary material for: Optimization of Alkaline and Dilute Acid Pretreatment of Agave Bagasse by Response Surface Methodology
Source: Front Bioeng Biotechnol. 2015 Sep 23;3:146. doi: 10.3389/fbioe.2015.00146 (PMC4585156; doi:10.3389/fbioe.2015.00146)
Supplement: Supplementary file 1 [file Table_1.DOCX]

**Table SI. Summary of thermal events of untreated and selected pretreated agave bagasse samples by DSC.**

| **Sample** | **Run** | **Thermal event 1** | | | **Thermal event 2** | | | |
| --- | --- | --- | --- | --- | --- | --- | --- | --- |
|  |  | **Onset (°C)** | **End**  **(°C)** | **ΔH**  **(J/g °C)** | **Onset (°C)** | **End**  **(°C)** | **Peak**  **(°C)** | **ΔH**  **(J/g °C)** |
| Untreated | - | 83 |  | 8.6 | 335 | 395 | 368 | 418.2 |
| AL-1 | 8 | 188.1 | 81.4 | 5.3 | 281 | 369 | 332 | 353.7 |
| AL-2 | 9 | 76 |  | 10.2 | 302 | 379 | 345 | 513.6 |
| AL-3 | 14 | 176 |  | 7.8 | 262 | 370 | 331 | 609.5 |
| AL-4 | 16 | 75 |  | 13.9 | 288 | 366 | 335 | 627.4 |
| DA-1 | 8 |  | 236.9 | 12.2 | 338 | 415 | 367 | 120.2 |
| DA-2 | 9 | 131 | 178 | 4.2 | 310 | 390 | 358 | 296.4 |
| DA-3 | 14 | 78 |  | 7.0 | 327 | 390 | 358 | 290.6 |
| DA-4 | 16 |  |  | 7.6 | 308 | 408 | 360 | 283.2 |
