# Supplementary figures and images for: Optimization of Alkaline and Dilute Acid Pretreatment of Agave Bagasse by Response Surface Methodology
Source: Front Bioeng Biotechnol. 2015 Sep 23;3:146. doi: 10.3389/fbioe.2015.00146 (PMC4585156; doi:10.3389/fbioe.2015.00146)

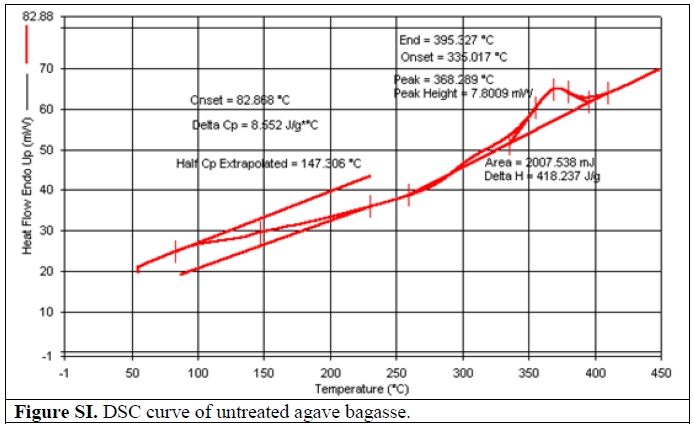

Supplement: Supplementary file 2 [file Image_1.JPEG]

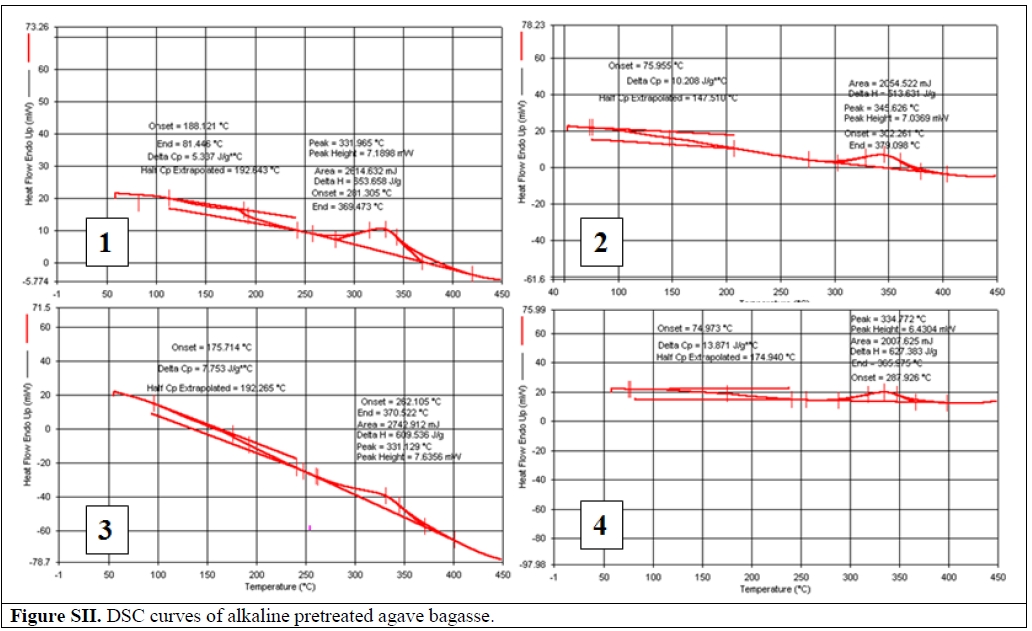

Supplement: Supplementary file 3 [file Image_2.JPEG]

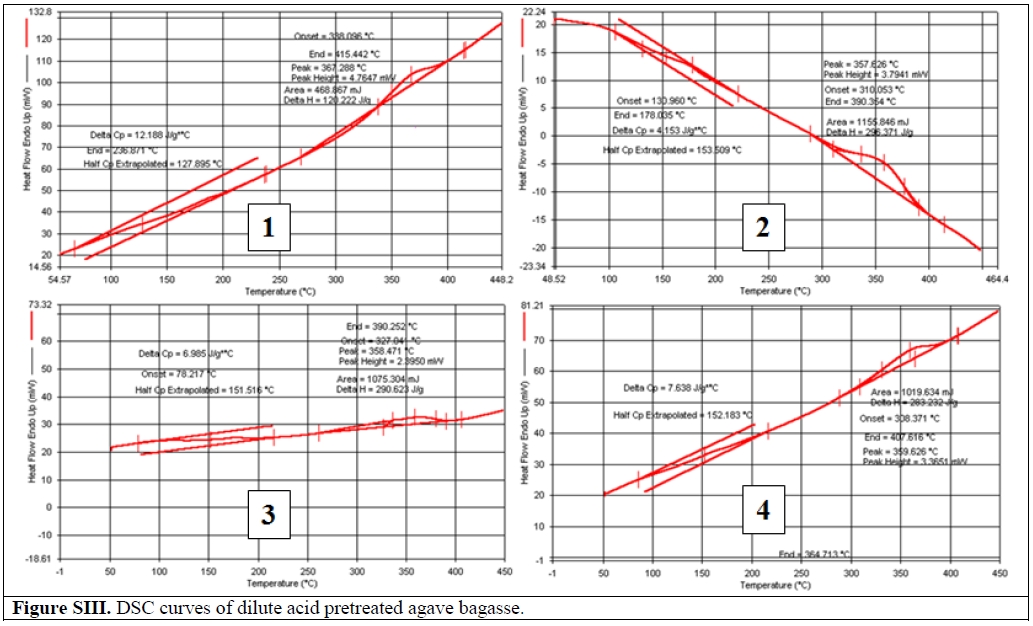

Supplement: Supplementary file 4 [file Image_3.JPEG]
